# Accelerated partial breast irradiation in early stage breast cancer

**DOI:** 10.3389/fonc.2022.1049704

**Published:** 2022-11-10

**Authors:** Paulina E. Galavis, Camille Hardy Abeloos, Pine C. Cheng, Christine Hitchen, Allison McCarthy, Juhi M. Purswani, Bhartesh Shah, Sameer Taneja, Naamit K. Gerber

**Affiliations:** Department of Radiation Oncology, New York University (NYU) Langone Health, School of Medicine, New York, NY, United States

**Keywords:** early stage breast cancer, accelerated partial breast irradiation, dosimetric considerations, treatment planning, toxicity outcomes

## Abstract

Accelerated partial breast irradiation (APBI) is increasingly used to treat select patients with early stage breast cancer. However, radiation technique, dose and fractionation as well as eligibility criteria differ between studies. This has led to controversy surrounding appropriate patients for APBI and an assessment of the toxicity and cosmetic outcomes of APBI as compared to whole breast irradiation (WBI). This paper reviews existing data for APBI, APBI delivery at our institution, and ongoing research to better define patient selection, treatment delivery, dosimetric considerations and toxicity outcomes.

## Introduction

Breast cancer is the most common cancer in American women with more than 250,000 invasive breast cancer diagnosed in the United States in 2021 (1). Since 2007, breast cancer mortality rates have continued to decrease in women over 50 years old with more than 3.8 million survivors in the United States (1). Standard of care for patients with early stage breast cancer after breast conserving surgery is radiation and endocrine therapy. As patients with breast cancer live longer, it is increasingly important to improve radiation delivery in order to minimize radiation sequelae.

Accelerated partial breast irradiation (APBI) focuses higher doses of radiation during a shorter time interval to the lumpectomy cavity rather than the whole breast. Different radiation techniques have been studied in phase III trials including multicatheter interstitial brachytherapy, balloon catheter intracavitary brachytherapy, external beam radiation therapy and intra-operative radiation therapy (2–5). **Table 1** summarizes these key trials (2–5, 7–14).

**Table 1:** Summary of key APBI trials.

Study	Years of enrollment	No patients/FU	Eligibility invasive	Eligibility DCIS	Dose Fractionation	IBTR	Toxicity
Hungary Polgar 2013 (3)	1998-2004	*N* = 258 10.2 yrs	pT1 (≤ 2), pN0–1mi, negative margins, age >40	Excluded	36.4 Gy/7 fx (brachytherapy) or 50 Gy/25 fx (electrons) vs 50 Gy/25 fx WBI	5.9% vs. 5.1%	PBI had higher excellent-good cosmetic score (81% vs. 63%)
Barcelona Rodriguez 2013 (4)		*N* = 102 5.0 yrs	pT1–2 (≤3 cm), pN0, margins ≥ 2 mm, age ≥ 60	Excluded	37.5 Gy/10 fx vs 48 Gy/24 fx WBI	0% Vs 0%	No difference in late skin toxicity or cosmesis
GEC-ESTRO Strnad 2016 (6)	2004-2009	*N* = 1,184 6.6 yrs	pT1–2 (<3 cm), pN0–1mi, margins ≥ 2 mm, age ≥ 40	Included with margin pure DCIS ≧̸ 5 mm	32 Gy/8 fx or 30.2 Gy/7 fx (HDR) or 50 Gy (PDR) Vs 50 Gy/25 fx WBI	1.4 vs. 0.9%	Reduced breast pain, less late grade 2–3 skin toxicity in APBI arm
IMPORT LOW Coles 2017 (7)	2007-2016	*N* = 2,018 6.0 yrs	pT1-2 (<3 cm), N0–1, margins ≥ 2 mm, age ≥ 50	Excluded	40 Gy/15 fx WBRT vs. 36 Gy WBRT+40 Gy APBI vs. 40 Gy/15 APBI	1.1% vs. 0.2% vs. 0.5%	Reduced toxicity in both experimental arms
NSABP B-39 Vicini 2019 (5)	2005-2018	*N* = 4,216 10.2 yrs	pT1–2 (<3 cm), pN0–1 (1-3), negative margins, age ≥ 18	Included	38.5 Gy/10 fx BID (3D), 34 Gy/10 fx BID (brachy) Vs 50 Gy/25 fx WBI	3.9% vs. 4.6%	APBI: grade 3: 10%, no grade 4–5 WBI: grade 3: 7%, no grade 4 or 5
RAPID Whelan 2019 (8)	2006-2018	*N* = 2,135 8.6 yrs	pT1–2 (≤ 2 cm), pN0-1mic, negative margins, age ≥ 40	Included	38.5 Gy/10 fx BID Vs 50 Gy/25 fx WBI	3% vs. 2.8%	Reduced acute and more late toxicity (grade 2+), similar patient rated cosmetic outcome in APBI arm
Florence Meattini 2020 (9)	2005-2013	N = 520 10.7 yrs	pT1–2 (< 2.5 cm), negative margins, age > 40	extensive DCIS excluded	30 Gy/5 fx QOD Vs 50 Gy/25 fx WBI	2.5% vs. 3.7%	Reduced acute and late toxicity and improved patient and physician rated cosmetic outcome in APBI arm
ELIOT Veronesi 2013 (10) Orecchia 2021 (11)	2000-2007	N = 1305 12.4 yrs	Age 48–75, pT1-2 (≤2.5 cm)	Excluded	21 Gy/1 fx IORT (prescribed to 90% IDL using 3–12 MeV electrons) Vs 50 Gy/25 fx WBI	11% Vs 2%	Reduced acute skin toxicity in IORT arm
TARGIT-A Vaidya 2020 (12)	2000-2012	N = 2298 5 yrs	Age ≥45, ≤3.5 cm, cN0-N1,	Included	20 Gy/1 fx IORT (o cavity surface (~5–7 Gy at 1 cm) with 50 kV photons) Vs WBI 3-6 weeks	2.1% Vs 0.95%	Reduced radiotherapy toxicity in IORT arm
ASTRO guidelines 2017 (13, 14)			pT1 (≤ 2 cm), pN0–1mi, margins ≥ 2 mm, age ≥ 50	screen-detected, 1-2 nuclear grade, ≤ 2.5 cm size, margins ≥ 3 mm			

NSABP B-39/RTOG 0413 is the largest prospective randomized trial completed to date, with over 4,300 patients with stage 0–II (≤3 cm) breast cancer or ductal carcinoma *in situ* (DCIS) status post lumpectomy with negative margins and 0–3 positive lymph nodes randomized to whole breast irradiation (WBI) (50 Gy with optional 10 Gy tumor bed boost) vs. APBI *via* either multicatheter brachytherapy (34 Gy in 10 fractions BID), intracavity brachytherapy (MammoSite 34 Gy in10 fractions BID), or 3D conformal radiation (3D-CRT) (38.5 Gy in10 fractions BID) (5). The 10-year cumulative incidence of in breast tumor recurrence (IBTR) was 4·6% (95% CI 3·7-5·7) in the APBI group versus 3·9% (3·1-5·0) in the WBI group. While APBI did not meet the criteria for equivalence to WBI, the absolute difference in IBTR was < 1%. Furthermore, the trial had broad eligibility criteria with a heterogeneous pool of patients and APBI techniques and was not designed to test equivalence in patient subgroups or outcomes from different APBI techniques.

The GEC-ESTRO trial randomized 1,184 patients to interstitial brachytherapy (32 Gy in 8 fractions or 30.2 Gy in 7 fractions) or WBI (50 Gy) and showed no difference in IBTR, 0.9% vs 1.4% respectively (6). Two large trials randomized patients to intraoperative radiation therapy (IORT) or WBI (10–12). In a cohort of 1,305 patients aged 48 to 75 y/o with unicentric tumors <2.5 cm s/p quadrantectomy the ELIOT trial showed higher rates of IBRT with IORT vs WBI: 11% vs 2% at median follow up of 12.4 years (p < 0.0001) (11). In the IORT arm, patients received 21 Gy/1 fx prescribed to 90% IDL using 3–12 MeV electrons). In a cohort of 2,298 patients ≤45 y/o with clinically unifocal IDC, the TARGIT-A trial showed no statistically significant difference between WBI and immediate IORT for local recurrence (12). In the IORT arm, patients received 20 Gy to cavity surface (~5–7 Gy at 1 cm) with 50 kV photons. Current ASTRO guidelines do not recommend low-energy IORT outside of prospective studies, while electron beam IORT is restricted to suitable risk patients.

The RAPID trial utilized APBI by 3D-CRT (38.5 Gy in 10 fractions BID), and found no difference in IBRT but an increase in moderate late toxicity and adverse cosmesis with APBI compared to WBI (8). However, the Barcelona trial using 3D-CRT and similar fractionation to the RAPID trial showed > 75% of patients in the APBI arm had excellent or good cosmesis and these outcomes were stable over time at a median follow up of 5 years (4). The Florence trial, which randomized patients to WBI vs. APBI using intensity modulated radiation therapy (IMRT) with 30 Gy in 5 every other day fractions, showed equivalent outcomes with APBI as compared to WBI and statistically significant less acute and late toxicity and improved cosmetic outcomes with APBI (vs. WBI) at a median follow up of 10 years (9). The IMPORT LOW trial randomized over 2000 patients to WBI, reduced-dose WBI with partial breast boost, and partial breast irradiation, all over 15 fractions, and showed no difference in IBTR with reduced toxicity with partial breast irradiation (7). Of note, in contrast to the other trials discussed, the IMPORT LOW trial was not an accelerated regimen as the fractionation was identical in the whole breast and partial breast arms.

## APBI at NYU

Patients have received APBI since 2000 at our institution (**Table 2**). The first patients at NYU treated with APBI were based on a pilot study conducted at the University of Southern California published in 2002 by Formenti et al. in which nine post-menopausal patients with pT1N0 breast cancer were treated in the prone position to 25 to 30 Gy in 5 fractions over 10 days using 5-7 horizontal photon treatment fields with couch rotations. Fractionation was based on biologically equivalent dose (BED) calculation for normal tissue (fibrosis, cosmesis) and tumor control (15). All patients were alive and disease free with good to excellent cosmetic results at follow up (median 41 months, range 36-53 months). Given the outcome of the USC pilot study, NYU 00-23 Hypo-Fractionated Conformal Radiation Therapy to the Tumor Bed after Segmental Mastectomy Phase I/II study was conducted between June 2000 and September 2007 (16, 17). 99 patients were treated in the prone position to 30 Gy in 5 fractions over 10 days using opposed mini-tangent photon fields (≥ 4MV). Treatment late toxicity assessment as per LENT (late effect of normal tissue)/SOMA (Subjective, Objective, Management, Analytics) showed low (1%) grade 2-3 toxicities (17) and reduced toxicity to organs at risk (16). Also, at 5 year follow up [95% level of confidence] the reported overall disease-free survival was 95% [87-98%] (17).

**Table 2:** NYU Partial Breast Development.

Study	Years of enrollment	No. Patients/FU	Target Definition	Dose Fractionation	Target Dose Constraints	OARs Dose Constraints	Planning	Imaging
USC Feasibility Pilot Study (15)	1997 - 1998	N = 9 41 months	PTV= lumpectomy cavity + 2 cm	25 - 30 Gy in 5 fxs over 10 days	D95% = 100% Dmax ≤ 110%	None reported	5-7 fixed horizontal 4 MV beams with couch rotations, avoiding non-breast tissue	Daily portal orthogonal images + two treatment fields
NYU 00-23 Hypo-Fractionated Conformal Radiation Therapy to the Tumor Bed After Segmental Mastectomy (Phase I/II Study) (16, 17)	2000 - 2007	N = 98 64 mos	CTV= lumpectomy cavity PTV= CTV + 2 cm limited anteriorly by skin and posteriorly by chest wall PTV Eval = PTV cropped 0.5 cm from skin surface and excluding chest wall	30 Gy in 5 fxs over 10 days (Mon, Wed, Fri, Mon, Wed)	PTV Eval: Covered by 95% of Rx dose (minimally 90%) Dmax ≤ 110%	Ipsilateral Breast: D50% < 50% of Rx dose Heart: no beam directed at heart Ipsilateral Lung: no beam directed at ipsilateral lung Contralateral Breast: no beam directed at contralateral breast	Opposed mini-tangents ≥ 4 MV	Daily portal images of each treatment field
NYU 07-582 Image Guided Radiotherapy (IGRT) For Prone Partial Breast Irradiation (PBI) (18, 19)	2007 - 2014	N = 297	CTV= lumpectomy cavity PTV = CTV + 1.5cm PTV Eval = PTV cropped to be within ipsilateral breast tissue, excluding first 0.5 cm of tissue under the skin, and tissue beyond the chest wall, pectoralis muscles, and lung	30 Gy in 5 fxs over 5 consecutive days	PTV Eval: D95% = 100% of Rx dose	Ipsilateral breast: V50% <60%; V100% < 35% Ipsilateral lung: V30% < 15% Contralateral lung: V5% < 15% Heart: V5% < 5%	3D or IMRT ≥ 4 MV No beam directed towards contralateral breast, heart or lung Non-coplanar beams encouraged, but not required	Day 1: CBCT and portal images Days 2-4: daily portal images Day 5: CBCT and portal images
NYU 14-01306 Prone Partial Breast Irradiation (PBI): Prospective Randomized Controlled Non-inferiority Trial To Compare Radiation Fibrosis With Five Versus Three Fractions	2014 - 2021	N = 284	CTV = lumpectomy cavity PTV = CTV + 1.5 cm PTV Eval = PTV cropped to be within ipsilateral breast tissue, excluding first 0.5 cm of tissue under the skin, and tissue beyond the chest wall, pectoralis muscles, and lung	Arm 1: 30 Gy in 5 fxs over 5 consecutive days Arm 2: 24 Gy in 3 fxs every other day	D95% = 100% of Rx dose	Ipsilateral breast: V50% < 60%; V100% < 35% Ipsilateral lung: V30% < 15% Contralateral lung: V5% < 15% Heart: V5% < 5%	3D or IMRT > 4 MV No beam directed towards contralateral breast, heart or lung Non-coplanar beams encouraged, but not required	Day 1: CBCT and portal images Subsequent days portal images kV images may be used to verify setup

NYU 07-582 Image guided Radiotherapy (IGRT) For Prone Partial Breast Irradiation (PBI) study was conducted between 2007 and 2014. 297 post-menopausal patients with pT1 breast cancer excised with negative margins were treated in the prone position to 30 Gy in 5 fractions over 5 consecutive days using 3D or IMRT fields. Wen et al. compared patients treated between 2003-2009 under NYU 0023 and NYU 07-582 with RTOG-0413 (5, 18). In RTOG 0413, patients were treated supine, CTV was defined as the cavity plus 1.5 cm expansion and PTV was defined as CTV plus 1.0 cm expansion (5). In NYU 00-23, patients were treated prone with the CTV defined as the lumpectomy cavity with no expansion and the PTV defined as CTV plus 2.0 cm expansion (16, 17). In NYU 07-582, patients were treated prone with CTV defined as the lumpectomy cavity with no expansion and the PTV defined as CTV plus 1.5 cm expansion (18). The main observation was that even though our PTV was 1 to 1.5 cm smaller than that of RTOG 0413 and our patients were treated prone as opposed to patients treated supine in RTOG 0413, our dosimetric results complied with the RTOG constraints for partial breast irradiation (18). A retrospective analysis of setup variations for 70 patients treated under NYU 07-582 confirmed adequacy of our CTV defined as lumpectomy cavity only and PTV defined as CTV plus 1.5 cm (19).

NYU 14-01306 Prone Partial Breast Irradiation (PBI): Prospective Randomized Controlled Non-inferiority Trial to Compare Radiation Fibrosis with Five Versus Three Fractions study was conducted between 2014 and 2021. 284 post-menopausal patients with pT1 breast cancer excised with negative margins received either 30 Gy in 5 fractions over 5 consecutive days (Arm 1) or 24 Gy in 3 fractions given every other day (Arm 2), using 3D-CRT or IMRT fields. Patients with lobular histology, Estrogen-receptor (ER) negative disease, and lymphovascular invasion (LVI) were included whereas those with an extensive intraductal component (EIC) were excluded. Pure DCIS was initially excluded but the trial eligibility criteria were later amended to include low risk DCIS as defined by the ASTRO APBI guidelines (13, 14).

## APBI at NYU – CT simulation, contours, beams, planning constraints and imaging

### CT simulation

Most of our APBI patients are simulated and treated in the prone position. We use the ClearVue™ prone breast board overlay (placed on sim and linac couch tops) which allows 18 cm free vertical hang space between board surface and base, and requires the head turned to the side. The kVue™ prone breast board (inserted into linac couch top) is selected for patients with pendulous breasts exceeding 18 cm vertical hang and/or for patients who prefer a neutral head position.

The patient is positioned prone on the breast board with the entire targeted breast hanging within the opening of the board. The sternal marker is palpated to ensure it is at the edge of the opening of the breast board. The contralateral breast is moved away from the targeted breast. A foam wedge is placed under the ankles for comfort.

Triangulation and lower alignment BBs and tattoos are placed. An additional mark on the lateral aspect of the breast in the axial plane of triangulation and a lower alignment mark on the lower back in the sagittal plane of posterior triangulation are used for isocenter location and alignment, respectively. The surgical scars are marked with radiopaque wire.

CT scan is acquired using 3 mm slice thickness, with upper and lower scan limits approximately at mastoid process/base of skull and 8 cm below the inframammary fold, respectively.

The simulation documentation includes the longitudinal scale value corresponding to the plane of triangulation, as well as the sagittal laser position corresponding to the posterior triangulation mark

**Figure 1:**
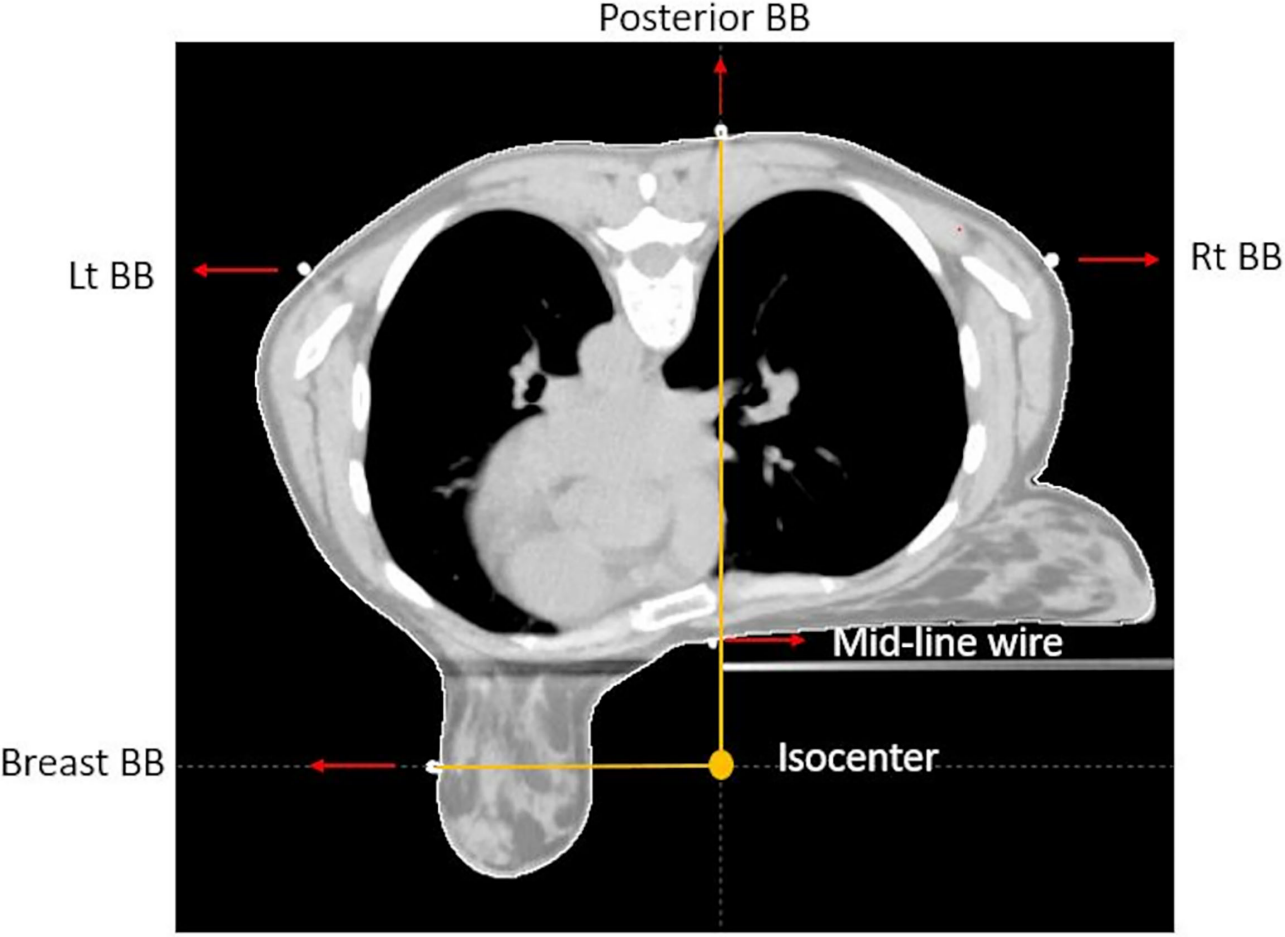
CT axial view of prone breast, showing isocenter location, triangulation point, lateral aspect of the breast marker, and midline marker.

When we simulate a patient prone for APBI, markers (Beekley RT-SPOT^®^ and CT SPOT^®^) are placed on the skin corresponding to midline along the sternum, lumpectomy incision, 2 cm inferior to the inframammary fold, and nipple (**Figure 1**).

### Contours

The tumor bed volume for each patient is drawn by the physician to include the resection cavity and any surgical clips (if placed). Pre-surgical imaging such as mammography and MRI is used to help delineate the tumor bed and attention is also given to the location of the scars marked at time of simulation. This volume is expanded to planning target volume (PTV) using a 1.5 cm uniform margin. The planning target volume evaluation (PTV_Eval) is the planning target volume (PTV) limited to be within the defined ipsilateral breast tissue, specifically excluding the 1st 5 mm of tissue under the skin and tissue beyond the chest wall, pectoralis muscles and lung. For all cases, the tumor bed volume, ipsilateral and contralateral breast are contoured by a physician. The other normal structures and OARs, including the ipsilateral and contralateral lung, heart and skin, are contoured by the dosimetry staff and reviewed by a physician.

### Beams

**Figure 2:**
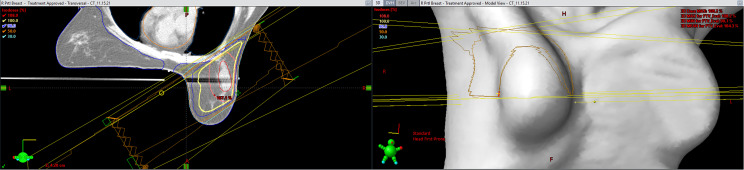
A representation of a typical external beam accelerated partial breast irradiation (APBI) plan in the prone position (Eclipse; Varian Medical Systems, Palo Alto, CA).

Treatment plans are generated in the Eclipse planning system (Varian Medical Systems) by a dosimetrist and reviewed by a physicist and physician. All patients are primarily treated using opposed photon tangents (3D-CRT or IMRT) in the prone position using a prone breast board with right- or left-sided apertures. The gantry and table angle combinations are selected to not enter or exit through other organs of the body. **Figure 2** includes a representation of a typical external beam APBI plan in the prone position. Patients treated in the supine position were planned using a similar technique with the possible addition of enface electrons.

### Constraints

The ipsilateral breast is constrained to V50% (V15 Gy) < 50-60% and V100% (V30 Gy) < 35%. Other constraints include heart V5% < 5%, ipsilateral lung V30% < 15%, contralateral lung V5% < 15%, PTV_Eval D95% > 100% and D99.5%>90%, Tumor Bed D98%>100%, and Body D0.03cc<110% (**Table 3**).

**Table 3:** Dose constraints for our APBI approach.

Structure	Constraint
Tumor Bed	D98% > 100%
PTV_Eval	PTV D95% > 100%.
	D99.5% > 90%
Body	D0.03cc < 110%
Breast – Ipsilateral	V50% (V15Gy) <50-60%
	V100% (V30 Gy) < 35%
Heart	V5% < 5%
Lung - Ipsilateral	V30% < 15%
Lung - Contralateral	V5% < 15%

### Imaging

On day 1: Cone-beam computed tomography (CBCT) image align to cavity, or breast if cavity is not visualized, then followed by MV portal images (no shifts are made based on MV images). For subsequent fractions only CBCT.

## Toxicity, dosimetry and outcomes of NYU patients 2010-2019

A retrospective study of 345 patients treated with APBI between 2010-2019 was performed, with 14 excluded due to APBI given for ipsilateral breast tumor recurrence (n=3), palliation (n=9), and incomplete RT course (n=2) (20). All patients being treated on NYU S14 01306, which was accruing at the time were also excluded. We did include patients who were on NYU 07-582 (60% of patients). Of the 331 total patients, the median age was 70 and 7.2% had DCIS. Of the 93% with invasive cancer, 9.8% had lobular histology, 2.3% were ≥ T2 stage, 0.9% were estrogen receptor negative, 1.3% had EIC, 2.6% had LVI, 0.3% were node positive, and 3.6% were multifocal. Margins were negative (using consensus criteria) in 67% of DCIS and 90% of invasive patients. In terms of RT delivery, 94% of patients were treated prone, with 32% treated every other day and 68% on consecutive days.

At a median follow-up of 5 years, there were 7 (2.1%) IBTR, 9 (2.7%) contralateral recurrences, and 1 (0.3%) distant metastasis. Five-year locoregional free survival was 99.5%, disease free survival was 96.7%, and overall survival was 98.1% (**Figure 2**). The 7 patients who experienced IBTR had unifocal pT1 tumors that were ER-strongly positive without EIC, LVI, or positive margins. When comparing those with IBTR (n=7) to those without (n=324), a higher proportion did not receive endocrine therapy (71.4% vs. 26.2%, p = 0.018). No differences were observed in other factors such as lobular histology (p = 0.49), margin status (p = 0.60), EIC (p = 1.0), LVI (p = 1.0), or ER negative disease (p = 1.0).

Rates of acute grade 1-2 dermatitis, fatigue and pain were 35.4%, 21.8% and 9.4% respectively, with no grade 3 toxicity (**Table 2**). The rate of good-excellent physician- and patient-rated cosmesis (n=199, median follow-up 2.8 years) was 92.5% and 89.4%, respectively. Patients experienced low rates of telangiectasia (4.5% grade 1 & 1.5% grade 2), fibrosis (17.6% grade 1 & 3.0% grade 2), and retraction/atrophy (24.1% grade 1, 2.5% grade 2, and 0.5% grade 3).

The mean PTV D95% was 100.0%. With regard to organs at risk, the average mean heart dose was 23.8 cGy for left-sided breast cancers and 12.7 cGy for right-sided breast cancers. Average ipsilateral lung V10% was 1.0% and V30% was 0.4%. In patients whose ipsilateral breast dose volume histogram (DVH) data were available (n=111), the mean ipsilateral breast V50% and V100% were 40.4% and 20.7%, respectively. These are further detailed in **Table 4**.

**Table 4:** DVH characteristics of patients treated from 2010-2019.

Parameter	Mean	Standard Deviation	Range
PTV D95% (%)	100.0	1.1	84.7-103.3
Mean Heart Dose (cGy)			
Left-Sided Tumors	23.8	0.5	0.0-81.0
Right-Sided Tumors	12.7	0.2	0.0-33.0
Heart V3Gy (%)			
Left-Sided Tumors	0.46	2.8	0.0-33.9
Right-Sided Tumors	0.0006	0.003	0.0-0.03
Ipsilateral Lung V10% (%)	1.0	2.2	0.0-22.0
Ipsilateral Lung V30% (%)	0.4	0.9	0.0-8.2
Ipsilateral Breast V50% (%)*	41.1	14.0	0.0-67.3
Ipsilateral Breast V100% (%)*	20.3	8.7	0.0-38.1

n=331 for all categories except those marked with (*), where n = 329.

DVH, dose volume histogram; PTV, planning target volume.

## Future directions

Given the variability in APBI technique, dose and fractionation, cosmetic outcome compared to WBI remains controversial. At a median follow-up of 10 years, NSABP-B39 reported higher grade 3 common toxicity criteria for adverse events in WBI arm vs APBI arm, 7% vs 10% respectively (5). Patient-rated cosmesis was equivalent but physician rated cosmesis was worse with APBI (21). We await more detailed publication of the toxicity, cosmesis and quality of life from this trial. The Canadian RAPID trial showed a statistically significant increase in late radiation toxicity in the APBI group primarily due to an increase in grade 2-3 breast fibrosis or induration (22.9% grade 2-3 induration or fibrosis in APBI group vs 4.6% in WBI) (8). However, the Barcelona trial using 3D-CRT and similar fractionation to the RAPID trial showed > 75% of patients in the APBI arm had excellent or good cosmesis and these outcomes were stable over time at a median follow up of 5 years (4). The Florence trial showed statistically significant less acute and late toxicity and improved cosmetic outcomes with APBI at a median follow-up of 10 years (9). We await the results of NYU S14 01306 which will report a 2 year rate of grade ≥ 2 fibrosis and long-term toxicity and cosmetic outcome. This trial is expected to meet its primary endpoint for all patients in June 2023.

Eligibility criteria for APBI has varied in clinical trials (**Table 1**). Initially, DCIS was largely excluded from APBI trials. NSABP B39, RAPID and Florence trials did include DCIS (5, 8, 9). In the current ASTRO guidelines, APBI is suitable for DCIS if it is screen detected, low-intermediate grade, ≤ 2.5 cm and margins ≥ 3 mm (13, 14). Age has also been a variable criteria. While current ASTRO guidelines consider APBI suitable for patients ≥ 50 years old and cautionary for patients 40-49 years old, Polgar et al., GEC-ESTRO trial, RAPID trial and Florence trial all included patients > 40 years old (3, 6, 8, 9, 13, 14). The size of the primary tumor, biological subtype, nodal status and definition of negative margins has also varied between trials. Given these variations, current ASTRO guidelines are currently being revised to better define eligibility criteria for APBI.

Finally, with the publication of the UK FAST FORWARD trial and increasing use of 5-fraction WBI fractionation schemes, the accelerated nature of APBI becomes less specific to partial breast and available for whole breast regimens as well (22). Thus future trials comparing APBI to accelerated WBI are necessary and the comparison of toxicity and cosmesis between APBI and WBI may shift as WBI schemes change.

## Author contributions

All authors listed have made a substantial, direct, and intellectual contribution to the work, and approved it for publication.

## Conflict of interest

The authors declare that the research was conducted in the absence of any commercial or financial relationships that could be construed as a potential conflict of interest.

## Publisher’s note

All claims expressed in this article are solely those of the authors and do not necessarily represent those of their affiliated organizations, or those of the publisher, the editors and the reviewers. Any product that may be evaluated in this article, or claim that may be made by its manufacturer, is not guaranteed or endorsed by the publisher.
